# Air pollution, epigenetics, and asthma

**DOI:** 10.1186/s13223-016-0159-4

**Published:** 2016-10-19

**Authors:** Hong Ji, Jocelyn M. Biagini Myers, Eric B. Brandt, Cole Brokamp, Patrick H. Ryan, Gurjit K. Khurana Hershey

**Affiliations:** 1 Division of Asthma Research, Cincinnati Children’s Hospital Medical Center, 3333 Burnet Ave. MLC 7037, Cincinnati, OH 45229 USA; 2 Pyrosequencing lab for Genomic and Epigenomic research, Cincinnati Children’s Hospital Medical Center, Cincinnati, OH 45229 USA; 3 Division of Biostatistics and Epidemiology, Cincinnati Children’s Hospital Medical Center, Cincinnati, OH 45229 USA

**Keywords:** Asthma, Traffic pollution, Epigenetics

## Abstract

Exposure to traffic-related air pollution (TRAP) has been implicated in asthma development, persistence, and exacerbation. This exposure is highly significant as large segments of the global population resides in zones that are most impacted by TRAP and schools are often located in high TRAP exposure areas. Recent findings shed new light on the epigenetic mechanisms by which exposure to traffic pollution may contribute to the development and persistence of asthma. In order to delineate TRAP induced effects on the epigenome, utilization of newly available innovative methods to assess and quantify traffic pollution will be needed to accurately quantify exposure. This review will summarize the most recent findings in each of these areas. Although there is considerable evidence that TRAP plays a role in asthma, heterogeneity in both the definitions of TRAP exposure and asthma outcomes has led to confusion in the field. Novel information regarding molecular characterization of asthma phenotypes, TRAP exposure assessment methods, and epigenetics are revolutionizing the field. Application of these new findings will accelerate the field and the development of new strategies for interventions to combat TRAP-induced asthma.

## Background

A recent comprehensive and systematic review of worldwide traffic emissions and health science by a special panel convened by the Health Effects Institute (HEI) found sufficient evidence that exposure to traffic-related air pollutants (TRAP) causes asthma exacerbation in children [1] and more recent reports have corroborated this [2, 3]. Within the complex mixture of gaseous and particulate components of TRAP, diesel exhaust particles (DEP) are of particular concern with respect to health effects. DEP are estimated to contribute up to 90 % of the particulate matter (PM) derived from traffic sources, are primarily ultrafine in size (<100 nm), can be deposited in the nasal and peripheral airways, and have been shown to induce oxidative stress and airway hyper-responsiveness, enhance allergic responses and airway inflammation [4–6]. This exposure is highly significant because in large cities in North America, up to 45 % of the population resides in zones that are most impacted by TRAP [1] and over 30 % of schools are located in high TRAP exposure areas [7]. Similar trends have been reported globally [8, 9]. Evidence from our group and others suggests TRAP is also associated with reduced lung growth and the development of asthma, though recent studies have reported conflicting results [10–16]. These inconsistent findings may be due to a lack of knowledge regarding the mechanistic basis of TRAP health effects and the characteristics of those most susceptibility to the harmful effects of TRAP exposure. Recent studies have started to fill gaps in knowledge regarding the molecular mechanisms by which TRAP leads to adverse effects on allergic diseases such as asthma. These studies demonstrate that exposure to DEP induces changes in DNA methylation that may have long lasting effects on health and future health risk.

### Epidemiology of the health impact of TRAP on allergic disease

The prevalence and incidence of allergic diseases, including asthma, have been increasing worldwide since the 1960s [17, 18]. While asthma prevalence has plateaued in developed countries, in developing countries where the prevalence was previously low, allergic diseases are on the rise [19]. Environmental changes are suspected to be the major driver of this increasing trend [20], with air pollution identified as an important exposure [21]. Motor vehicles produce a complex mixture of air pollutants including carbon monoxide, nitrogen oxides, particulate matter (PM) of varying size, polycyclic aromatic hydrocarbons (PAHs—e.g. benzo(a)pyrene), volatile organic compounds (VOCs—e.g. benzene, acetaldehyde) and other hazardous air pollutants (HAPs). Collectively referred to as traffic-related air pollutants (TRAP), these are the primary source of intraurban variability in air pollutant concentrations [1].

There is sufficient evidence to suggest that TRAP can decrease lung function and trigger asthma exacerbation and hospitalizations [18, 22]. Recent large studies on TRAP and respiratory outcomes substantiate these conclusions. Findings from the University of Southern California’s Children’s Health Study (CHS), a cohort of 11,365 schoolchildren in 16 communities, indicate that exposure to higher local nitrogen dioxide (NO_2_) concentrations and close residential proximity to freeway increase asthma prevalence [23]. Asthmatic children in the cohort that lived in communities with higher levels of NO_2_, PM_10_ and PM_2.5_ had increased chronic lower respiratory symptoms, phlegm, production, bronchitis, wheeze and medication use [23]. In Korea, children aged 6–14 (n = 5443) living within 200 m of a main road that was ≥254 m long had increased lifetime wheezing, lifetime asthma diagnosis and decreased lung function [24]. A meta-analysis of six cohorts in the European Study of Cohorts for Air Pollution Effects (ESCAPE) that included 23,704 adults found that exposure to higher NO_2_ increased the incidence of adult-onset asthma, although the results did not reach significance [25].

Birth cohort studies have evaluated the impact of TRAP on asthma development in children. In the Cincinnati Childhood Allergy and Air Pollution Study (CCAAPS) birth cohort, a child’s risk for persistent wheeze and asthma development varied depending on the timing and duration of TRAP exposure [26]. The TRAP exposure level at the child’s birth address was associated with wheezing [27–29] and recurrent night cough [30] in the first 3 years of life. Children exposed to high levels of TRAP at birth were nearly twice as likely to experience persistent wheezing at age seven; however, a longer duration of exposure to high levels of TRAP (beginning early in life and continuing through age seven) was the only time period of exposure related to asthma development [26].

The ESCAPE project is comprised of five birth cohort studies including 17,041 children. While these birth cohorts did not find any significant associations between six traffic-related pollution metrics and childhood asthma prevalence, the land-use regression (LUR) models used to estimate exposures were carried out as long as 15 years after the asthma outcomes were collected [14]. During this time, campaigns to reduce air pollution could have reduced exposure levels compared to those present when the asthma outcomes were collected.

In 2015, Bowatte et al. conducted a systematic review and meta-analysis of birth cohort studies to understand the association between early childhood TRAP and subsequent allergies, asthma and allergic sensitization [16]. While significant associations were observed between asthma incidence and PM_2.5_ and black carbon (BC), there was substantial heterogeneity observed (likely due to differences in study design, participants and exposure and outcome definitions) between the studies [16]. Nevertheless, their review highlights that traffic-related air pollution (TRAP) is associated with new onset of asthma throughout childhood, and the authors suggest that TRAP exposure may have an ongoing effect with a lag time of about 3 years [16].

Reduced lung function as a consequence of air pollution exposures is also a recognized risk factor for long-term respiratory effects. The ESCAPE Project found that estimated levels of NO and PM were associated with small but significant reductions in lung function in school children [31]. Most recently, the investigators from the Child Heart and Health Study in England (CHASE) evaluated the effects of air pollution on lung function in children both in their cohort and in a systematic review and meta-analysis that included the ESCAPE studies [32]. In CHASE, they observed that residential levels of oxides of nitrogen and PM showed inverse but non-significant association with both FEV_1_ and FVC [32]. When the CHASE results were included in a meta-analysis of published studies, a statistically significant association between NO_2_ and FEV_1_ was observed. The authors estimate that every 10 μg/m^3^ increase in NO_2_ is associated with a 0.7 % decrease in FEV_1_, which translates into a 7 % increase in the prevalence of children with abnormal lung function [32], which is a significant public health concern. Similar to the CHASE meta-analysis, in 1968 Latino and African-American children from the US and Puerto Rico, a 5 μg/m^3^ increase in average lifetime PM_2.5_ was associated with a 7.7 % decrease in FEV_1_ [33]. In children aged 10–18 participating in the University of Southern California CHS mentioned above, living within 500 m of a freeway was associated with a significant reduction in FEV_1_, FVC and maximal mid-expiratory flow rate compared to those living more than 1500 m away [23].

Collectively, there is considerable evidence that TRAP plays a role in the development, and/or symptoms of asthma. However, heterogeneity in both the definitions of TRAP exposure and asthma outcomes and unmeasured confounding limit the ability to draw firm conclusions from the data. As discussed in the Bowatte et al. meta-analyses, there is substantial variability in the exposure measurements across TRAP-related studies. Land use regression (LUR) models are among the most common methods to assess TRAP exposures [16]. Other methods include passive samplers, central monitoring stations and proximity to major roads. The most frequent markers of pollutants include PM, oxides of nitrogen, carbon monoxide and ozone. PM may be further reported as BC, PM_10_, or PM_2.5_. While this vast variation in the definition of TRAP exposure limit the ability to conduct sound meta-analyses, it highlights the importance of appropriate exposure assessment, as discussed below.

The other central challenge is the vast heterogeneity of asthma. The term “asthma” encompasses a number of distinct phenotypes of asthma, which have different molecular signatures. These asthma “endotypes” are subsets of disease defined by a distinct functional or pathobiological mechanisms [34]. The linkage to pathogenic mechanisms makes recognition of endotypes especially valuable, as knowledge of pathogenic mechanisms of specific variants of asthma may serve as a more precise guide to treatment. TRAP-induced asthma is a distinct phenotype of asthma, which was recently shown by our group to be characterized by increased levels of serum IL-17A in children and increased CD4^+^IL13^+^IL17^+^ double-producing T effector memory cells in mice [6, 35]. Thus, studies of the health effects of TRAP exposure need to carefully define and characterize both the exposure variable and the health outcome.

### Assessment of TRAP exposure

Given the increasingly evident health impact of TRAP, methodologies to accurately assess exposure are needed. While TRAP affects air quality on urban and regional scales, their impact is greatest on a local scale, particularly near roadways, as their concentrations are significantly elevated within approximately 300–500 m of their source [36]. Further influencing individuals’ TRAP exposure is its temporal variability combined with complex and variable personal behavior including time spent indoors/outdoors [37]. In order to meet the intrinsic challenge of accurately assessing TRAP exposure for epidemiologic studies both modeling and personal measurement approaches have been utilized. Because particulate matter (PM) is a complex mixture of chemical and elemental constituents, recent studies have focused on assessing exposure and associating health effects with specific elemental PM components, rather than the more traditionally used total PM mass. Most notably, the large ESCAPE project has developed land use regression models for particle composition in twenty study areas in Europe [38]. Accurate and precise models were built for individual components and the group used these to associate exposure to PM2.5 nickel and sulfur with decreased lung function in five cohorts of children [39]. Furthermore, they found that long term exposure to PM2.5 copper and PM10 iron was associated with increased levels of inflammatory blood markers [38].

While regulatory air monitoring provides valuable data to link regional and temporal variability of air pollutants to population-level health outcomes [40–43], these networks are unable to capture the high spatial variability of TRAP concentrations within an urban area. Measuring proximity (i.e. distance) to major roadways is a straightforward approach to estimate TRAP exposure, though this method does not account for traffic density and other geographic and land-use characteristics which impact TRAP concentrations [44]. Dispersion models have also been used to assess exposure to TRAP, but this approach has been limited to a small number of locales with available emissions and meteorology data required for this approach [10, 45, 46].

The most frequently used method to estimate TRAP exposure in epidemiologic studies is land use regression (LUR) modeling [44, 47–49]. In the most straightforward LUR approach, a single pollutant from the TRAP mixture is measured at multiple stationary sites within a defined study region and characteristics of the area surrounding each sampling site (e.g. elevation, nearby roads, traffic) are used as predictors of the measured concentrations in a linear model. The resultant LUR model is then applied to estimate pollutant concentrations at non-sampled locations including schools and homes where significant geographic predictor variables can be determined [14, 15, 44, 48, 50–57]. Recently, research groups have created land use models to predict the concentration of individual components of PM in more urban environments [58]. The temporal variability of TRAP concentrations have also been incorporated into LUR models through the addition of mobile or continuous monitoring allowing for short-term and daily estimates of TRAP exposure for study participants [52, 59–62]. LUR models have also been shown to accurately capture the spatial variability in pollutant concentrations over a period of 7 or more years [63, 64]. New data inputs for LUR models, including satellite-derived pollutant measurements [65, 66] and the development of hybrid models combining LUR with Bayesian Maximum Entropy have also improved the accuracy of TRAP exposure assessment [67, 68]. In studies with available participant-reported time spent in locations outside the home, LUR models have been used to derive time-weighted estimates of exposure based on location [48]. More recent application of this time-weighted approach have utilized smartphones and GPS-derived location data to improve estimates of TRAP exposure by combining LUR or other modeled TRAP estimates with individuals’ location through space and time [69]. External model validation is also key to accurate exposure assessment. Researchers recently found that models developed for specific neighborhoods were not generalizable to other neighborhoods, but that a general model that was locally calibrated performed similarly to neighborhood-specific models [70].

Despite advances in modeling TRAP and the incorporation of GPS to improve estimates of individual-level exposure, personal monitoring remains the ‘gold-standard’ for TRAP exposure assessment. The use of mobile monitoring has increased in popularity, in part due to its ability to cover a higher spatial resolution as compared to stationary monitoring. Land use regression models have been developed using data from mobile laboratories [71] as well as cars and bikes [72], allowing for resolutions up to 20 m. Although the increased resolution is an advantage of using mobile monitoring to collect air pollution measurements, it requires multiple repeated measurements to precisely predict exposure.

### Mechanistic insights into TRAP effects on the epigenome and the pathogenesis of asthma

Although there is strong evidence that TRAP exposure contributes to childhood asthma [1, 10, 11, 29], the mechanistic basis of TRAP effects on asthma has been elusive. The epigenetic, molecular, and cellular pathways triggered by exposure to TRAP and their impact on allergen-induced immune responses have been studied in human studies as well as in reductionist models in vitro and in animal models in vivo.

#### The basics of epigenetics

The concept of epigenetics keeps revolving since Waddington first coined the word to describe mechanisms that regulate gene expression and contribute to development [73]. A modern definition of an epigenetic trait is a stably heritable phenotype resulting from changes in a chromosome without alterations in the DNA sequence [74]. To date, epigenetic mechanisms include DNA methylation, histone modification, histone variants, nucleosome positioning, non-coding RNA and other newly discovered phenomenon such as RNA methylation. Together, these epigenetic mechanisms regulate the gene expression programs of a cell by being responsive to changes in the environment of a cell, including all the developmental signals and environmental cues that lead to diseases, which is particularly evident during cellular differentiation and cancer development [75–77]. A compelling hypothesis is that environmental cues associated with diseases might initiate or influence the epigenetic processes of host cells, leading to epigenetic reprogramming of host cells to favor their pathogenic function and contributing to the development of the disease. DNA methylation is the first epigenetic mechanism recognized and most extensively studied in human populations. Thus, the main focus of this part of the review is on DNA methylation, its association with air pollution and asthma, and its impact on the association between air pollution with asthma.

**Fig. 1:**
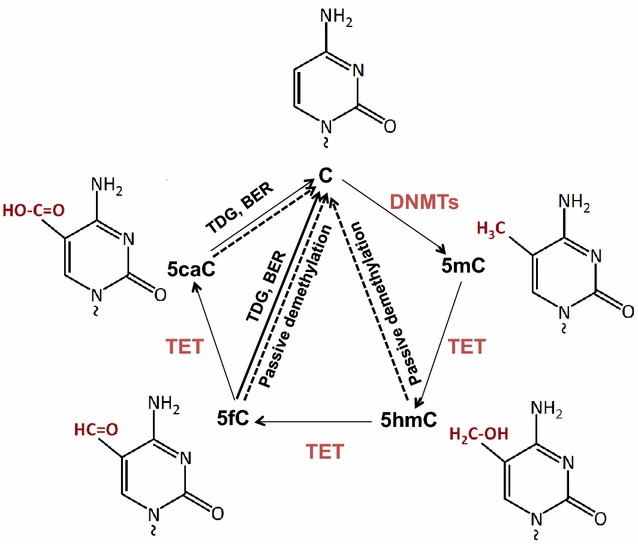
DNA methylation and demethylation in mammals. DNMTs methylate cytosine C to 5-methylcytosine (5mC) by transferring the methyl group from S-adenosylmethionine (SAM) to cytosine. TET enzymes oxidize 5mC to 5-hydroxymethylcytosine (5hmC), 5-formylcytosine (5fC), 5-carboxylcytosine (5caC) (together, oxi-mC). Further, oxi-mC can be restored to C through the thymine DNA glycosylase (TDG)-mediated base excision repair (BER) of 5fC:G and 5caC:G base pairs and replication-dependent passive demethylation

DNA methylation is the chemical modification of cytosine by covalently adding a methyl group to its 5′ carbon (5-methylcytosine, or 5mC), which is mostly found in the context of CpG dinucleotide. CpG islands (defined as regions of more than 200 bases with a G + C content of at least 50 % and a ratio of observed to an expected frequency of at least 0.6) are often found at functionally relevant genomic elements, such as promoters and enhancers, indicating their important role in gene regulation. Genome-wide DNA methylation profiling by next-generation sequencing in several species has demonstrated that DNA methylation at the promoter and at the 3′ end of a gene is negatively associated with gene expression levels, whereas whole gene body methylation seems to be positively associated with gene expression levels [78, 79]. In mammalian cells, DNA methylation is maintained through the coordinated actions of DNA methyl-transferases (DNMTs), which catalyze the transfer of a methyl group from S-adenosyl methionine (SAM) to the carbon 5′ position of cytosine (Fig. 1). Replication of symmetrically methylated CpGs leads to hemi-methylated parent-daughter duplexes, which will be methylated by DNMT1/UHRF1 complex [80, 81]. Non-CpG methylation occurs primarily in pluripotent stem cells and neuron cells [82, 83], and is maintained by two de novo methylases, DNMT3a and 3b [83, 84]. Recently TET proteins (ten-eleven translocation family) were identified as dioxygenases that utilize two key factors Fe(II) and 2-oxyglutarate (2-OG), to oxidize the methyl group of 5mC to hydroxylmethyl, formyl, or carboxyl groups [85–89] (Fig. 1). The resulting oxi-mC intermediates (5hmC, 5fmC and 5caC) can be restored to C by active or passive mechanisms [88, 89], resulting in DNA demethylation (Fig. 1).

Many cellular differentiation processes, including immune cell differentiation, are accompanied by dynamic changes in DNA methylation and other epigenetic changes, which often occur at key transcription factors sites and at genomic locations encoding functional molecules such as cytokines to control their lineage commitment [76, 90–92]. Protein components in epigenetic machinery such as DNMTs, TETs and DNA methyl-group binding proteins, often bind to these cytokine signature gene loci through interaction with key transcriptional factors, setting up local epigenomic structure and controlling their expression [92]. In addition, environmental cues including air pollution can directly regulate the expression levels of DNMTs and TETs [93–96], or accumulation of these enzymes at targeted genes [97, 98], therefore modify the epigenomic landscape of key genes involved in asthma pathogenesis.

#### DNA methylation and asthma

Epigenomic regulation of T cell differentiation plays an important role in the process of allergic sensitization [99–101], including T helper cell differentiation (Th1, Th2 and Th17) and the establishment of regulatory T cell phenotype (Treg). Activation of the T helper 2 (Th2) type cytokine profile is a hallmark of experimental asthma. Epigenetic remodeling including DNA methylation changes and histone modifications has been shown to influence Th2 polarization and associated cytokines and chemokines involved in the development of asthma [102–104, 106]. Further, pharmacological modification of *IFNγ* methylation in T cells modifies asthma phenotypes in animal models [107]. Tregs also play an essential role in allergic responses in asthma [108] and *FOXP3* is the master regulator of Tregs. The regulation of *FOXP3* expression by methylation at its proximal promoter and an intronic regulatory element is well-established and studies from twins discordant for asthma indicate that this mechanism is important for asthma development [99, 109].

Although asthma has long been characterized as a disease of dysregulated T_H_2 immune responses to environmental allergens, accumulating evidence suggests a role for T_H_17 cells, especially severe steroid resistant asthma. Serum IL-17A is significantly higher in severe asthmatics compared to mild asthmatics or controls in adults and children [110–112]. Recent studies have demonstrated that dual-positive T_H_2/T_H_17 cells and IL-17A were present at a higher frequency in the bronchoalveolar lavage fluid (BALF) from steroid resistant asthmatic patients [113]. These T_H_2/T_H_17 cells were resistant to dexamethasone-induced cell death and the T_H_2/T_H_17 predominant subgroup of patients manifested the most severe form of asthma [113]. As an important player in asthma development, especially air pollution-related asthma [6], the epigenetic regulation of Th17 cells is not well understood. Recently it has been shown that the promoter of *IL17a* and intron 2 of *Rorα* (IL17 associated transcriptional factor) were demethylated in ex vivo isolated murine Th17 cells and in murine Th17 cells generated invitro compared to naïve T cells and other T helper cells [114]. This is consistent with a previous report highlighting the epigenetic regulation of Il17a and IL17f expression by promoter DNA methylation and histone modifications in in vitro generated murine Th17 cells [115].

In addition to genes implicated in T cell function, which have been relatively well studied, association studies in human populations identified epigenetic variations in other genes important for asthma, including those involved in immune responses, nitric oxide synthesis, lipidomics, and pharmacologic receptors [99]. In support of these previous findings, a recent report using a genome-wide approach identified an IL13-induced DNA methylation signature in adult asthmatic airways, which contains two co-methylation modules related to asthma severity and eosinophilia respectively [116]. Using the same platform, another study compared blood DNA methylation levels between 97 controls and 97 inner city asthmatic patients and identified 81 differentially methylated CpG sites [117]. Validated CpG sites are located at *RUNX3* (related to T cell maturation), *IL4* (related to Th2 function), and *catalase* (related to oxidative stress). CpG sites associated with serum IgE among asthmatics were also discovered in this study. An epigenome-wide association study also identified and validated 36 CpG sites whose methylation levels in blood DNA are significantly associated with serum IgE level (FDR < 10^−4^) [118]. Importantly, the top three CpG sites account for 13 % of IgE variation, which is tenfold higher than that derived from large single nucleotide polymorphism (SNP) genome-wide studies. This implies a significant role for epigenome in asthma and underscores that the epigenome may be a rich source of novel biomarkers for asthma and potentially new targets for asthma therapy. Recent evidence from our group associated lower *TET1* promoter methylation and higher 5hmC levels in airway epithelial cells with childhood asthma, uncovering a novel role of *TET1* and DNA demethylation in asthma development [95]. In addition, researchers also started to look at DNAm markers for asthma that develops in childhood and persists into early adulthood [119] and markers for temporal asthma transition [120]. Despite these investigations, DNAm variations consistently associated with asthma are rarely found, possibly due to differences between cohorts, definition of asthma phenotypes, and from which tissue DNAm is measured. Disease-epigenetic variation is often tissue-specific, which should be accounted for when interpreting the results. How this should be considered in epigenomic epidemiologic studies has been discussed in other reviews [121].

Studies of DNA methylation are often coupled with gene expression studies and genetic variation studies, as DNA methylation can regulate gene expression [122] and SNPs also modify DNA methylation [123, 124]. Morales et al. demonstrated an interaction between SNPs within *ALOX12* and a nearby DNA methylation variation that is significantly associated with childhood wheezing in three cohorts [125]. Interestingly, this interaction is most evident for those SNPs tagged by rs312466, and rs312466 is ~300 away from the interacting CpG site, indicating an in *cis* interaction. In the Swedish birth-cohort BAMSE, Acevedo and colleagues studied the association of childhood asthma with SNPs, regional DNA methylation, and gene expression at the *GSDMB/ORMDL3* locus located at 17q21, a well-studied asthma-susceptibility locus found in ethically diverse populations [126]. They found that 3 SNPs that either created or removed CpG sites altered DNA methylation *in cis* and were associated with asthma. Methylation at these SNP-CpG sites was correlated with *ORMDL3* expression and associated with methylation levels at other CpG sites in this locus, including ones located in the *ORMDL3* promoter. They also found that the methylation levels in the *ORMDL3* promoter was higher compared to controls, and correlated with *ORMDL3* expression in blood leukocytes from asthmatic children. Together, these data suggest interactions among CpG sites and between CpG sites and SNPs within this locus in asthma. Future well-designed, integrative genome- and epigenome-wide associations studies are needed to examine the interplay between genetic and epigenetic factors and how these interactions contribute to asthma in a cell type-specific manner.

#### Air pollution and DNA methylation

The epigenome is postulated to be a mechanistic bridge between air pollution and the development of asthma, possibly via mediating gene-environment interactions [99, 127]. Indeed, combined inhaled diesel exhaust particles and allergen exposure in mice lead to changes in promoter methylation of the asthma related gene *IL4*, and methylation levels are correlated with serum IgE changes [128]. These findings are consistent with numerous studies that have demonstrated that exposure to either particulate matter or DEP exacerbates T_H_2 responses. One recent study using co-cultures of OVA transgenic CD4^+^ T cells and bone marrow derived dendritic cells (BMDC) pre-exposed to OVA with or without TRAP, demonstrated increased IFNγ, IL4, IL13, and IL17 levels in culture supernatants of OVA + TRAP exposed BMDC compared to BMDC exposed to OVA alone [129].

A growing body of literature has identified DNA methylation variations associated with different types of air pollution in human populations, including TRAP [127, 130]. TRAP is a mixture of carbon monoxide, nitrogen oxide, PM, PAH, VOCs and other HAPs. Among these components, PM_2.5_ from various sources has been associated with DNA methylation changes [130]. However, these studies are often inconsistent, possibly due to the differences in exposure measurement and different relative amounts of the TRAP components within the estimate; therefore, the impact of TRAP on repeat element methylation or global DNA methylation remains uncertain. One recent study evaluated the in vitro epigenotoxicity of six different types of ambient air PM [131] including soil dust, road dust, agricultural dust, biomass burning, traffic exhausts, and pollen. Indeed, these different types of PMs have very different, sometimes opposite, effects on the expression and enzymatic activity of DNMTs and the methylation of repetitive elements. Further, such impact is time-specific and dose-dependent.

Using a candidate gene approach, multiple cohort studies have consistently linked DNA methylation levels in the inducible nitric oxide synthase gene (*iNOS*) with exposure to particulate matter [132–137]. *iNOS* and other components in the nitric oxide synthase pathway are responsible for nitric oxide production, and children with asthma and allergic airway diseases have measurably higher fractional concentration of exhaled nitric oxide (FeNO) [138, 139]. Interestingly, an interaction between genetic variants, DNA methylation variation within *iNOS,* and PM exposure has been noted [137]. Other genes whose methylation levels in saliva DNA have been associated with ambient air pollution have also been implicated in asthma. A recent study identified an association between methylation of 31 genes and exposure to BC utilizing a pathway-based approach [140]. The genes included *HLA*-*DOB* (MHCII), *FCER1A* and *FECR1G* (IgE receptor), *IL9*, and *MBP* (eosinophil granule major basic protein), which are related to the Th2/B cell signaling pathway, eosinophils, and airway inflammation. Increased exposure to ambient air pollution was also associated with hypermethylation of *FOXP3*, which coincided with impaired Treg function and increased asthma morbidity [141]. Hypermethylation of *IFN*-*γ* in effector T cells was associated with increased exposure to ambient air pollution in the same cohort [142] and was further supported by observations from the Normative Aging Study [143]. Research from our group also uncovered the association of saliva *FOXP3* methylation with TRAP exposure during the 1st year of life and persistent wheezing and asthma diagnosis at age 7 in the CCAAPS cohort [144], which implicates the epigenome as a mediator of the impact of early life TRAP exposure on later asthma risk. Further work using the fast developing genome-wide approaches to identify TRAP-associated DNA methylation changes in relevant tissues using a longitudinal design are needed.

Recently we found that *TET1* promoter methylation is associated with both TRAP exposure and asthma prevalence in children [95]. Exposure of airway epithelial cells to DEP altered the expression of *TET1*, and resulted in changes in global 5hmC [95]. Further, exposure to PM_10_ was associated with higher global 5hmC levels over time, but not with global 5mC levels [145]. In the same cohort, PM exposure was associated with hypomethylation of selected tandem repeats, such as *NBL2* and *SATa* [146]. Taken together, these data support a role for 5hmC and *TET1* in response to TRAP exposure and highlight the need to differentiate 5hmC and 5mC in future environmental epigenetic studies.

To directly investigate the short-term DNA methylation changes induced by exposure to TRAP in humans, controlled exposure studies have been performed in adults [147, 148]. A cross-over study in 15 healthy adults showed that exposure to concentrated ambient particles (CAPs) for 130 min lowered methylation levels at specific loci [147]: fine CAPs exposure lowered *Alu* methylation, while coarse CAPs exposure lowered *TLR4* methylation. In another cross over study [148], sixteen non-asthmatic adults were exposed to diesel exhaust (300 **μ**g/m^3^ PM2.5) for 2 h on two separate occasions at least 2 weeks apart. RNA samples isolated from their peripheral blood mononuclear cells (PBMCs) were subjected to the Infinium HumanMethylation450 BeadChip array to identify genome-wide changes associated with DEP exposure at 6 h or 30 h after the second exposure. Genes encoding protein kinases and other proteins in the NF-kB pathways become less methylated after the exposure. In a more recent study [149], Clifford et al. conducted a randomized, crossover-controlled exposure study in which 17 adults were exposed to filtered air or diesel exhaust (DE, 300 μg/m^3 ^PM2.5) followed by saline via segmental allergen challenge. Genome-wide DNAm studies using bronchial epithelial cells collected 48 h after the last challenge identified changes at 6 CpG sites in response to DE, 7 sites in response to co-exposure (DE and allergen), while allergen alone didn’t cause any significant differences. Interestingly, allergen challenge 4 weeks after DE exposure induced DNAm changes at more CpG sites (75 sites at p < 0.05, difference in β > 0.10) suggesting that DE exposure may have long lasting effects on epigenetic responses to subsequent exposures.

Exposure to other components in TRAP, specially PAH, has also been associated with DNA methylation changes [130]. The epigenetic effects of PAH can begin in utero, which may lead to long-term health problems. Maternal exposure to PAH is associated with DNA methylation changes in the acyl-CoA synthetase long-chain family member 3 (*ACSL3*) gene in cord blood cells of children and is also associated with higher risk of developing asthma [150]. Maternal PAH exposure has also been linked to increased methylation of *IFN*-*γ* promoter in cord white blood cells [151]. Collectively, these data support the notion that methylation modifications can link in utero exposure with asthma development [152–154]. Similarly to PM exposure, exposure to ambient PAH can be associated with impaired Treg function and increased methylation of *FOXP3* [155].

**Fig. 2:**
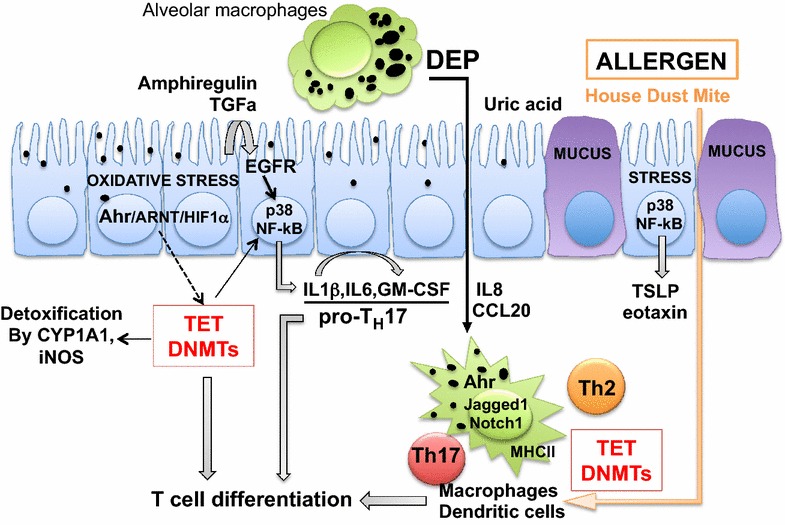
Epigenetic mechanisms mediate DEP effects on asthma pathogenesis. Lung epithelial cells recognize polycyclic aromatic hydrocarbons present in diesel exhaust particles (DEPs) via the aryl hydrocarbon receptor (AhR), promoting cytochrome P450 family 1 A1 (CYP1A1)-mediated and iNOS-mediated detoxification through altering methylation. Failure to detoxify results in oxidative stress, which may upregulate TETs and downregulate DNMTs through the crosstalk between AhR and HIF1-α and directly lead to secretion of chemokines (eosinophils/neutrophils) and cytokines involved in TH17 and TH2 differentiation (TSLP), Treg differentiation and B cell function, all contributing to airway inflammation. The secretion of these chemokines and cytokines can also be triggered by repair cytokines (amphiregulin, TGFα) signaling through the epidermal growth factor receptor (EGFR), p38 mitogen-activated protein kinase, and NF-κB, which can be augmented by demethylation and upregulation through TET proteins and DNMTs. DEP promotes allergic airway inflammation by upregulating the expression of the Jagged1/Notch1 pathway in dendritic cells (DC) in an AhR dependent manner in concert with allergens. DEP may also regulate DNMT and TET expression in dendritic cells and macrophages through the AhR pathway, enhancing airway inflammation in presence of allergens. *DEP* diesel exhaust particle; *OVA* ovalbumin; *TSLP* thymic stromal lymphopoietin

How TRAP, including DEP and PAH, modifies the epigenome remains unclear. One hypothesis is that the epigenetic changes are mediated via the AhR, which subsequently regulates the expression and function of the epigenetic machinery that can activate/repress target genes related to inflammation and immune responses (Fig. 2). It has been shown that the expression of DNMTs and TETs is altered in the lungs of asthmatics [93–95]. A few recent reports have demonstrated the regulatory role of TET1/2/3 in hematopoiesis [156], B cell lineage specification [157] and Treg differentiation [158–160], which are all involved in asthma development. Exposure to air pollution can regulate the expression levels of these proteins in airway epithelial cells and alveolar macrophages [95, 131] (Fig. 2). Air pollution may also modify the accumulation of epigenetic enzymes at genetic loci involved in asthma pathogenesis [97, 98], thereby modifying the local epigenomic landscape of these genes and contributing to asthma. Expression of DNMTs and TET1 can both be regulated in a HIF1α-dependent matter under hypoxia conditions [161, 162]. In addition, it is reported that oxidative stress and the generation of reactive oxygen species (ROS) can regulate HIF1α transcription in humans [163]. Since the HIF1α and AhR pathways may intersect [164], it is plausible that the effects of DEP are partially mediated through interactions between AhR, HIF1α, DNMTs and TETs (Fig. 2). Futures studies elucidating the interactions between AhR signaling and the epigenetic machinery are needed to pinpoint the mechanisms by which TRAP contribute to asthma.

#### The impact of early life TRAP exposure

The timing and duration of traffic-related air pollution (TRAP) exposure may be important for childhood wheezing and asthma development. High TRAP exposure at birth was significantly associated with wheezing phenotypes in a birth cohort, but only long-term exposure to high levels of TRAP throughout childhood was associated with asthma development [26]. Indeed, in the Cincinnati Childhood Allergy and Air Pollution Study (CCAAPS) birth cohort, early TRAP exposure was associated with persistent wheeze while early and sustained exposure to TRAP was associated with asthma development [26]. As discussed above, saliva *FOXP3* methylation was found to be associated with TRAP exposure during the 1st year of life and persistent wheezing and asthma diagnosis at age 7 in the CCAAPS cohort [144], suggesting that the epigenome may contribute to the impact of early life TRAP exposure on later asthma risk.

Prenatal TRAP exposure has been linked to asthma as well [165–168]. Mothers who lived near highways during pregnancy are more likely to have children with asthma [166]. Prenatal exposure to PAHs is associated with increased risk of allergic sensitization and early childhood wheeze [165, 168]. A limited number of mechanistic studies have assessed the impact of in utero TRAP exposure on the development of allergic disorders. In one recent study, offspring of mice exposed to DEP were hypersensitive to OVA and developed increased OVA sensitization, airway inflammation, Th2/Th17 responses, and AHR compared to offspring with no prior in utero DEP exposure [169]. Further, prenatal DEP exposure induced expression of genes downstream of AhR and this upregulation persisted 1 month after birth, even though mice were no longer exposed to DEP. Thus, in utero DEP exposure appears to result in a primed state where the neonate is hypersensitive to subsequent allergen exposure. In mice exposed to ambient particulate air pollution near steel mills and major high ways, there is significant, persistent sperm DNA hypomethylation [170], suggesting a transgenerational effect of TRAP exposure. Thus, the epigenetic changes induced by TRAP may have very long lasting effects. While the epigenetic mediation of the trans-generational impact of numerous exposures (endocrine disruptors, high fat diets) is being actively explored; the evidence for the epigenetic mediation of trans-generational effects of TRAP is lacking and in need of better investigation.

## Conclusion

As discussed above, there is considerable evidence that exposure to TRAP is associated with childhood asthma development, symptoms, and exacerbations. Herein, we have reviewed the recent findings regarding the epigenetic mechanisms by which TRAP exposure mediates its negative health effects. These findings have identified potential biomarkers that could enable rapid and reliable identification of individuals at-risk due to high exposure in the future. Further, new methodologies for quantification of TRAP will enable accurate assessment of exposure in real time such that interventions could be designed and implemented early in the course of exposure in vulnerable populations. Additional studies are needed to fill the remaining gaps including more careful characterization of the epigenetic modifications and the upstream/downstream pathways, the study of interactions between genetic and epigenetic variations, the impact of the timing, load, and duration of TRAP exposure on the durability of the epigenetic modifications, and translation of these findings to clinical applications.
